# Aryl Hydrocarbon Receptor Agonist VAF347 Impedes Retinal Pathogenesis in Diabetic Mice

**DOI:** 10.3390/ijms22094335

**Published:** 2021-04-21

**Authors:** Thomas E. Zapadka, Sarah I. Lindstrom, Julia C. Batoki, Chieh A. Lee, Brooklyn E. Taylor, Scott J. Howell, Patricia R. Taylor

**Affiliations:** 1 Department of Ophthalmology and Visual Sciences, School of Medicine, Case Western Reserve University, Cleveland, OH 44106, USA; tez9@case.edu (T.E.Z.); sxl926@case.edu (S.I.L.); jxb986@case.edu (J.C.B.); cxl77@po.cwru.edu (C.A.L.); taylo465@miamioh.edu (B.E.T.); sjh36@case.edu (S.J.H.); 2 Louis Stokes Cleveland VA Medical Center, Cleveland, OH 44106, USA

**Keywords:** diabetic retinopathy, AhR, VAF347, retinal inflammation, capillary degeneration, IL-17A

## Abstract

Diabetic retinopathy is the leading cause of blindness in the working-age population worldwide. Although the cause of diabetic retinopathy is multifactorial, IL-17A is a prevalent inflammatory cytokine involved in the promotion of diabetes-mediated retinal inflammation and the progression of diabetic retinopathy. The primary source of IL-17A is Th17 cells, which are T helper cells that have been differentiated by dendritic cells in a proinflammatory cytokine environment. Aryl hydrocarbon receptor (AhR) is a ligand-dependent transcription factor that can manipulate dendritic cell maturation, halt the production of IL-6 (a proinflammatory cytokine), and suppress Th17 cell differentiation. In the current study, we examined the efficacy of an AhR agonist, VAF347, as a potential therapeutic for the onset of non-proliferative diabetic retinopathy in streptozotocin (STZ)-induced diabetic C57BL/6 mice. We determined that diabetes-mediated leukostasis, oxidative stress, and inflammation in the retina of STZ-diabetic mice were all significantly lower when treated with the AhR agonist VAF347. Furthermore, when VAF347 was subcutaneously injected into STZ-diabetic mice, retinal capillary degeneration was ameliorated, which is the hallmark of non-proliferative diabetic retinopathy in this diabetes murine model. Collectively, these findings provide evidence that the AhR agonist VAF347 could be a potentially novel therapeutic for non-proliferative diabetic retinopathy.

## 1. Introduction

With 463 million diabetics in the world, diabetes is one of the most prevalent non-communicable diseases worldwide [1,2,3]. Previous studies have provided evidence that diabetes mediates chronic, low-grade inflammation, which leads to vascular impairment in the heart, kidney, and retina [4,5,6]. Inflammation is a physiological response to infection, wherein cytokines such as Interleukin-17A (IL-17A) are produced at the mucosal site to clear the host of extracellular pathogens. In immune competent individuals, after the pathogen is cleared, only negligible levels of IL-17A are produced and inflammation subsides [7,8]. However, in diabetes, IL-17A is constantly produced and inflammation does not halt, which can lead to kidney failure, heart disease, or vision loss [4,5,6,9].

In the retina, these microvascular alterations can induce capillary non-perfusion and the onset of diabetic retinopathy, which is the leading cause of blindness in the working-age population worldwide [2,10]. Previously, we determined that diabetes induced IL-17A production, which enhanced retinal inflammation, oxidative stress, and retinal capillary degeneration in streptozotocin (STZ)-induced diabetic mice [11,12]. The most prevalent source of IL-17A is CD4^+^ T helper-17 (Th17) cells, which are RORγt^+^ T cells that have been differentiated by dendritic cells in a proinflammatory cytokine environment [13,14]. Recently, when diabetes-mediated IL-17A production was ablated by therapeutically inhibiting RORγt, retinal capillary degeneration was ameliorated and the onset of non-proliferative diabetic retinopathy was halted [15]. In our current study, we examined an aryl hydrocarbon receptor (AhR) agonist, VAF347, that can inhibit Th17 cell differentiation as another potentially novel therapeutic for diabetic retinopathy.

Aryl hydrocarbon receptor (AhR) is a ligand-dependent transcription factor that can be activated by aromatic hydrocarbons found in many different synthetic, dietary, and environmental products [16]. When activated, AhR translocates to the nucleus and binds to the dioxin- or xenobiotic-responsive element in the promoter site of monocytes and manipulates dendritic cell maturation and T cell differentiation [16,17]. By selectively expanding specific types of dendritic cells and regulating MHC-II expression during antigen presentation, AhR can halt the production of pro-inflammatory cytokines, enhance the proliferation of T regulatory (Treg) cells, and suppress Th17 cell differentiation [18,19]. Previous studies in other autoimmune disorders provide evidence that the AhR agonist VAF347 inhibited the production of proinflammatory cytokines and IL-17A, as well as suppressed Th17 cells while enhancing T regulatory (Treg) cell differentiation [20,21]. Taken together, we postulated that AhR agonist VAF347 will halt Th17 cell differentiation and IL-17A production, which will ameliorate retinal inflammation, and halt the onset of non-proliferative diabetic retinopathy in STZ-diabetic mice.

In this current study, we determined that when the AhR agonist VAF347 was therapeutically administered to diabetic mice, Th17 cells and IL-17A production was ameliorated. This significantly decreased leukostasis, oxidative stress, and retinal inflammation in STZ-induced diabetic mice, halting capillary degeneration and early-stage non-proliferative diabetic retinopathy. Taken together, these results suggest that VAF347 is a potentially novel therapeutic for the onset and progression of non-proliferative diabetic retinopathy.

## 2. Results

### 2.1. Hyperglycemia in STZ-Induced Diabetic Mice

Diabetes-mediated hyperglycemia was sustained throughout a two-month (*n* = 30/group, except +VAF347-ND (*n* = 10)) or an eight-month (*n* = 10/group) period in STZ-induced diabetic C57BL/6 mice. Fasted (6 h) blood glucose levels were measured 17 days after the last STZ-injection (day 22) to confirm diabetic conditions in untreated C57BL/6 mice, whereas all diabetic mice had an average blood glucose level of 500 mg/dL or greater. Sera were evaluated in both untreated (C57BL/6) and AhR agonist VAF347-treated (+VAF347) non-diabetic (ND) and STZ-diabetic (DB) mice to quantify average blood glucose levels through a glycated hemoglobin A1c (HbA_1c_) analysis at week 6 and 29 after diabetes was confirmed (Table 1). Both the diabetic untreated and the diabetic VAF347-treated mice had significantly higher A1c blood glucose levels than their non-diabetic controls at both 6- and 29-weeks post-diabetes. However, there was no significant difference amongst the A1c blood glucose levels of the untreated and VAF347-treated diabetic C57BL/6 mice (Table 1).

### 2.2. Injections of AhR Agonist VAF347 Inhibit Diabetes-Mediated IL-6 and IL-17A Production, and Systemically Ablates Th17 Cells in STZ-Diabetic Mice

**Figure 1:**
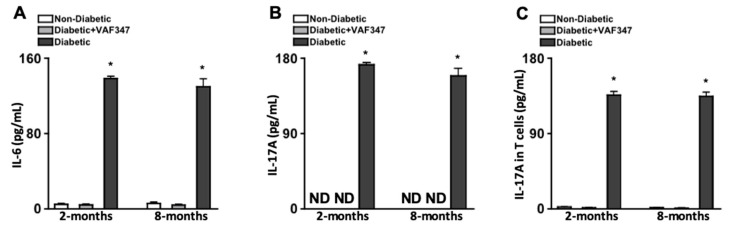
Levels of IL-6 and IL-17A in sera, and IL-17A in CD4^+^ T cells of STZ-diabetic mice. ELISA quantification of IL-6 (**A**) and IL-17A (**B**) in sera collected from non-diabetic (white, *n* = 5), VAF347-treated STZ-diabetic (grey, *n* = 5), and untreated STZ-diabetic (black, *n* = 5) C57BL/6 mice; two and eight months after diabetes was confirmed. (**C**) Quantification of IL-17A in protein lysates of CD4^+^ T cells isolated from spleens of non-diabetic (white), VAF347-treated STZ-diabetic (grey), and untreated STZ-diabetic (black) C57BL/6 mice two and eight months after diabetes was confirmed. * = *p* < 0.01 per two-way ANOVA and unpaired Student’s *t*-test. ND = not detected. Data are representative of two separate experiments with similar results.

Previously, it was determined that administering 30 mg/kg of a derivative of the AhR agonist VAF347 (VAG539) inhibited dendritic cell maturation and IL-6 production, while skewing T cell differentiation [21,22]. In this study, we administered 30 mg/kg of VAF347 in 100 μL subcutaneous injections after diabetes was confirmed. To determine if this treatment regimen was sufficient to inhibit IL-6 and IL-17A production, sera were collected from mice (*n* = 5) two and eight months after diabetes was confirmed, and the levels of IL-6 and IL-17A were quantified by ELISA. Negligible levels of IL-6 (Figure 1A) and no IL-17A (Figure 1B) was detected in the sera of non-diabetic mice (white) at both time points. However, at both two and eight months, ~140 pg/mL of IL-6 was detected in the sera of untreated STZ-diabetic mice (black), which was significantly decreased to negligible levels in the VAF347-treated STZ-diabetic (grey) mice (Figure 1A). Furthermore, ~175 pg/mL of IL-17A (Figure 1B) was detected in the sera of untreated diabetic mice (black), which was ablated in the VAF347-treated diabetic mice (grey).

To ascertain if the VAF347 therapeutic regimen was sufficient to ameliorate diabetes-mediated Th17 cell differentiation, spleens were collected from non-diabetic, diabetic, and VAF347-treated diabetic mice two and eight months after diabetes was confirmed (*n* = 3/group per time point), CD4^+^ T cells were isolated by negative selection columns, and the levels of IL-17A in T cell lysates was quantified by ELISA analysis. As shown in Figure 1C, no IL-17A was detected in the T cells of non-diabetic mice (white), yet ~150 pg/mL of IL-17A was detected in the untreated diabetic (black) which was ameliorated to undetectable levels of IL-17A in the T cells of the diabetic mice treated with VAF347 (grey). Taken together, these results suggest the VAF347 treatment regimen is sufficient to halt diabetes-mediated IL-17A and Th17 cell differentiation.

### 2.3. AhR Agonist VAF347 Treatment Significantly Decreases Leukostasis in STZ-Diabetic Mice

**Figure 2:**
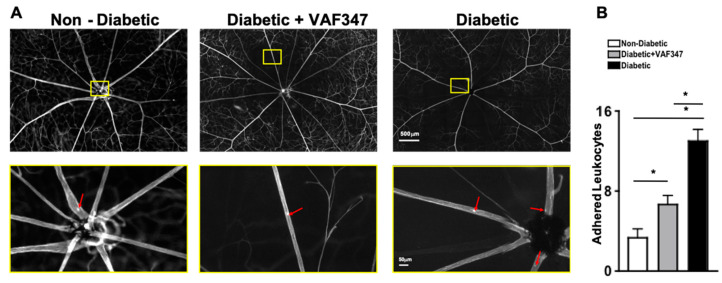
Leukostasis in the retinal vasculature of STZ-diabetic mice. (**A**) Representative images of leukostasis in the retinas of non-diabetic, VAF347-treated diabetic, and untreated diabetic C57BL/6 mice; following perfusion and staining. Total adherent leukocytes are shown in the upper quadrants, while the lower quadrant displays magnified sections, with red arrows highlighting leukocytes adhered to the retinal vasculature. (**B**) Quantification of the number of leukocytes adhered to the retinal vasculature in all mice (*n* = 3/group). Scale bars = 500 μm (upper quadrant) and 50 μm (lower quadrant) are displayed as a visual indicator of the size of the representative image. * = *p* < 0.01; *p*-value was first equated by two-way ANOVA and then an unpaired *t*-test with Tukey’s post-hoc analysis.

Leukocytes adhere to the vasculature wall (referred as leukostasis) in the retinas of diabetic mice. The adhesion of leukocytes to the capillary endothelium can lead to blood–retina barrier (BRB) breakdown, followed by vascular leakage, capillary non-perfusion, and the onset of non-proliferative diabetic retinopathy [23]. To determine if the AhR agonist VAF347 is capable of inhibiting leukostasis, FITC-stained adherent cells in flat mounts of retinas from non-diabetic, diabetic, and VAF347-treated diabetic mice were manually quantified (*n* = 3/group). Red arrows highlight adherent leukocytes in the retinal vasculature of non-diabetic, untreated STZ-diabetic, and VAF347-treated STZ-diabetic mice (upper quadrant, Figure 2A). Each representative image in the lower quadrant of Figure 2A is a magnification of a leukocyte adhered to the vessel wall in the retina. As shown in Figure 2A, there were very few leukocytes adhered to the vessel walls in the retina of non-diabetic mice, and a significantly higher number of adherent leukocytes in the vessels of the retinas of untreated STZ-diabetic mice (Figure 2A,B). Yet, leukostasis was significantly decreased in the retinas of diabetic mice that received the VAF347 treatment when compared to the untreated STZ-diabetic mice (Figure 2B). Although leukostasis was slightly higher in the retinal vasculature of the VAF347-treated diabetic mice than the non-diabetic mice, this was not a statistically significant increase. These results suggest that the AhR agonist VAF347 is sufficient to significantly decrease diabetes-mediated leukostasis, which can lead to capillary non-perfusion and the onset of non-proliferative diabetic retinopathy.

### 2.4. VAF347 Treatment Significantly Decreases Oxidative Stress and Retinal Inflammation in STZ-Diabetic Mice

**Figure 3:**
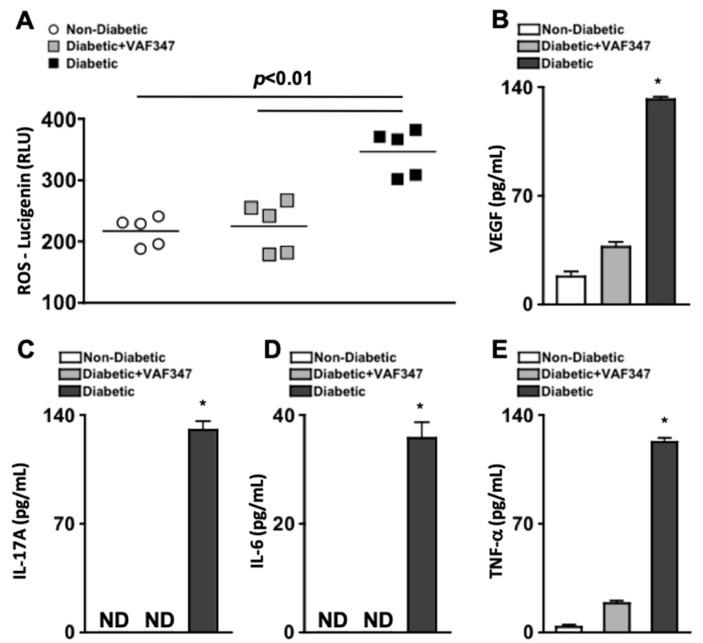
**Oxidative stress and retinal inflammation in STZ-diabetic mice.** (**A**) Quantification of reactive oxygen species (ROS) in the retinas of non-diabetic (white circles), VAF347-treated STZ-diabetic (grey squares), and STZ-diabetic (black squares) C57BL/6 mice; each data point represents an individual retina (*n* = 5/group). ELISA quantifications of VEGF (**B**), IL-17A (**C**), IL-6 (**D**), and TNF-α (**E**) in retinas (*n* = three samples of three pooled retinas/group) of non-diabetic (white), STZ-diabetic VAF347-treated (grey), and STZ-diabetic (black) C57BL/6 mice. ROS and inflammatory protein analysis were performed two months after diabetic conditions were confirmed. ND = not detected. * = *p* < 0.01 wherein *p*-value was first equated by two-way ANOVA analysis and then unpaired Student’s t-test. Data are representative of two separate experiments with similar results.

To test the efficacy of the AhR agonist VAF347 against retinal oxidative stress during diabetes, reactive oxygen species (ROS) was quantified two months after diabetic conditions were confirmed in STZ-diabetic mice. Levels of ROS were detected using lucigenin in the retinas of non-diabetic (white circles), VAF347-treated diabetic (grey squares), and untreated diabetic (black squares) C57BL/6 mice (*n* = 5/group). ROS was significantly increased in the retinas of untreated diabetic mice when compared to non-diabetic mice, which was significantly lowered when the diabetic mice were treated with VAF347 (Figure 3A). There was no significant difference in the levels of ROS in the retinas of diabetic mice treated with the AhR agonist VAF347 and the non-diabetic mice (Figure 3A).

Vascular endothelial growth factor (VEGF) is one of the most prevalent proinflammatory proteins induced by diabetic hyperglycemia and plays a pivotal role in diabetes-mediated vascular permeability, leakage, and proliferation in the retina [24]. Likewise, IL-17A, IL-6, and TNF-α are the primary cytokines involved in retinal inflammation, retinal vascular impairment, and the progression of diabetic retinopathy [11,25]. To evaluate the efficacy of AhR agonist VAF347 to inhibit retinal inflammation, retinal protein lysates were analyzed by ELISA. Retinas were collected and protein lysates isolated from non-diabetic (white), diabetic (black), and VAF347-treated diabetic (grey) C57BL/6 mice (*n* = three separate samples of three pooled retinas/group); two months after diabetic conditions were confirmed. Levels of VEGF, IL-17A, IL-6, and TNF-α were quantified by ELISA analysis. As shown in Figure 3B–E, no IL-17A or IL-6, and only negligible levels of TNF-α were detected in the retinas of non-diabetic mice. Approximately 20 pg/mL of VEGF was detected in the retinas of non-diabetic mice (Figure 3B). Conversely, ~135 pg/mL of VEGF, IL-17A, and TNF-α and ~40 pg/mL of IL-6 were detected in the retinas of untreated STZ-diabetic mice (Figure 3B–E). However, all inflammatory proteins were significantly decreased in the retinas of the VAF347-treated diabetic mice, whereas no IL-17A or IL-6 were detected (Figure 3C,D), and only ~20 pg/mL of VEGF (Figure 3B) and TNF-α (Figure 3E) were detected in the protein lysates of retinas. Collectively, this provides evidence that VAF347 can sufficiently halt diabetes-mediated retinal inflammation and oxidative stress in the retina, which is the precursor to diabetic retinopathy.

### 2.5. VAF347 Treatment in STZ-Diabetic Mice Halts Retinal Capillary Degeneration

**Figure 4:**
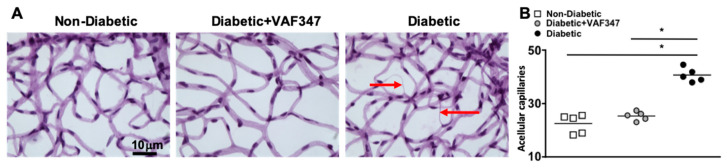
Capillary degeneration in the retinas of STZ-diabetic mice. (**A**) Representative images of acellular capillaries and degeneration in retinal capillary beds of non-diabetic, VAF347-treated STZ-diabetic, and untreated STZ-diabetic C57BL/6 mice (scale bars of all images = 10 μm). Red arrows highlight acellular capillaries. (**B**) Quantification of acellular capillaries within a 1.10 mm^2^ area of each retina in non-diabetic (white square), VAF347-treated STZ-diabetic (grey circle), and untreated STZ-diabetic (black circle) C57BL/6 mice. Each data point represents an individual retina from five different mice. *p*-value was first equated by two-way ANOVA analysis and then an unpaired *t*-test with Tukey’s post-hoc analysis, wherein * = *p* < 0.01. All samples were collected eight months after diabetic conditions were confirmed.

In the early stages of diabetic retinopathy and in this eight-month murine model, retinal endothelial cells die, causing acellular and degenerative capillaries. This is the first clinical sign of diabetic retinopathy [26,27]. To determine if the AhR agonist VAF347 treatment is sufficient to halt capillary degeneration in the diabetic retina, we isolated the capillary beds of retinas (*n* = 5) from non-diabetic, VAF347-treated STZ-diabetic, and untreated STZ-diabetic C57BL/6 mice, eight months after diabetes was confirmed. All acellular capillaries (representative examples are highlighted by red arrows in Figure 4A) were manually quantified. The number of acellular capillaries in the retinas of untreated STZ-diabetic mice was significantly higher than in non-diabetic C57BL/6 mice (Figure 4B). However, the number of acellular capillaries in the retinas of STZ-diabetic mice receiving VAF347 therapeutics was significantly decreased and similar to that of non-diabetic mice (Figure 4B). This indicates that VAF347 is sufficient to halt diabetes-mediated retinal endothelial cell death and capillary degeneration, which is a clinically relevant hallmark of non-proliferative diabetic retinopathy. Hence, these results suggest that the AhR agonist VAF347 would be a good therapeutic candidate for non-proliferative diabetic retinopathy.

## 3. Discussion

Inflammation is one of the leading causes of all diabetic complications, including diabetic retinopathy [28]. Hyperglycemic spikes continuously occur in diabetics that induce low-grade inflammation, which initiates leukostasis and asymptomatic alterations in the retinal microvasculature [25]. This leads to retinal inflammation, oxidative stress, and blood–retina barrier breakdown. Chronic retinal inflammation induces capillary degeneration and non-perfusion, as well as vascular permeability and leakage, which are the first clinically detectable signs of non-proliferative diabetic retinopathy. This vascular impairment initiates angiogenic signaling, which induces neovascularization in the retina, proliferative diabetic retinopathy, and vision loss [29,30,31,32]. Although laser treatments, steroids, and anti-angiogenic VEGF inhibitors have minimized vision loss, all of these therapeutics are administered in diabetics with late-stage proliferative diabetic retinopathy or diabetic macular edema. Currently, therapeutics for early-stage non-proliferative diabetic retinopathy is very limited. Nevertheless, our current study provides strong evidence that the AhR agonist-VA347 could be a good therapeutic candidate for early-stage non-proliferative diabetic retinopathy.

The cause of diabetic retinopathy is multifactorial, with many studies providing evidence that multiple proinflammatory proteins play a pivotal role in the progression and pathogenesis of diabetic retinopathy [11,12,24,25,33]. In our current findings, we discovered that the AhR agonist VAF347 not only inhibited diabetes-mediated VEGF and retinal inflammation, but also systemically ablated hyperglycemic induced IL-6 production. Previous studies provide evidence that IL-6 plays a role in the onset and progression of diabetic retinopathy. In both animal and human studies, IL-6 has been linked to an increased production of reactive oxygen species [34], retinal endothelial cell death [34,35], vascular permeability [35], and choroidal neovascularization [36]. In our current studies, we discovered that VAF347 significantly decreased reactive oxygen species and retinal endothelial cell death (acellular capillaries detected in Figure 4). It is feasible to suggest that the significant decreases in these retinal pathologies may be partially due to VAF347 inhibiting IL-6 production.

Alternatively, the therapeutic impact of the AhR agonist VAF347 may be due to its inhibitory effect on Th17 cell differentiation and IL-17A production. AhR plays a critical role in Th17, Th22, and Treg differentiation [18,19]. The AhR agonist-VA347 inhibits the differentiation of monocytes to CD86 and HLA-DR-expressing dendritic cells, which are relevant in Th17 cell differentiation [19]. This inhibitory activity can halt Th17 cell differentiation and IL-17A production. In our previous studies, we determined that Th17 cells adhered to the retinal vasculature in diabetic mice, which led to retinal inflammation, vascular leakage, and capillary degeneration [11,12]. In this current study, we discovered that leukostasis was significantly decreased in the retinal vasculature of diabetic mice treated with VAF347. Additionally, retinal inflammation and capillary degeneration were ameliorated in the diabetic mice that received VAF347 treatment. Collectively, these data suggest that the VAF347 inhibitory impact on both Th17 cell differentiation and IL-17A production played a pivotal role in this potential therapeutic, negating the onset of diabetic retinopathy.

The most eminent finding in this study was the therapeutic impact of VAF347 on the onset of non-proliferative diabetic retinopathy. However, it is also notable that this study further defines the role of Th17 cells in the pathogenesis of diabetic retinopathy. Previous studies provide evidence that diabetes mediates Muller glia to produce IL-17A, which plays a role in the onset and progression of diabetic retinopathy [37]. Until this study, the exact role of Th17 cells and IL-17-producing Muller glia was unclear. Since AhR is not expressed in Muller glia [38], and Muller glia are not differentiated by AhR-expressing dendritic cells, the AhR agonist VAF347 should only impact Th17 cells [16]. Hence, the retinal pathology that was ameliorated by VAF347 is the Th17-dependent retinal pathology. Future mechanistic studies need to be performed to fully delineate the role of Th17 cells in the onset and progression of diabetic retinopathy, which goes beyond the scope of this study. However, our current findings suggest that Th17 cells are the primary source of diabetes-mediated IL-17A, which enhance retinal pathology and the onset of non-proliferative diabetic retinopathy.

IL-17A and Th17 cells have been previously identified as pivotal components in the induction of autoimmune type I diabetes [39], initiation of obesity-driven type II diabetes and insulin resistance [40], and the onset of multiple diabetic complications [4,5,12]. However, in this STZ-induced diabetes model, we did not detect any difference in hyperglycemia or body weight after the mice received VAF347 treatment. This is probably due to the limiting parameters of this diabetes murine model, wherein the pancreatic beta cells are permanently damaged by the streptozotocin without the ability to recover metabolic function. The STZ-diabetes murine model is optimal for diabetic retinopathy studies because retinal pathogenesis progresses to retinal capillary degeneration, which is a clinically relevant hallmark of non-proliferative diabetic retinopathy. Yet, to further examine the therapeutic impact of VAF347 in the onset and progression of diabetes and hyperglycemia, another diabetes model would have to be used in future studies. Hence, it is still feasible that VAF347 could be used as a therapeutic for both diabetes and diabetic complications.

Since there is an FDA approved anti-IL-17A drug available, it is possible that this drug could be used to halt the progression of diabetes and its complications. However, anti-IL-17A is a monoclonal antibody that is too large to cross the blood–retina barrier. If anti-IL-17A was administered systemically, it could potentially halt the systemic progression of diabetes and some of its complications. However, since it cannot cross the blood–retina barrier, it would not provide any therapeutic impact for diabetic retinopathy. Alternatively, anti-IL-17A could be administered by intravitreal injections, but this would only provide treatment for retinal pathogenesis and diabetic retinopathy. Hence, a small molecule therapeutic that could be administered systemically, which could cross the blood–retina barrier and provide treatment for the retina as well as other organs impacted by diabetes would be the optimal therapeutic for diabetes and its complications. We suggest that these findings provide evidence that the AhR agonist VAF347 is capable of crossing the blood–retina barrier and can systemically ablate pathogenic Th17 cells and IL-17A. Accordingly, we propose that the AhR agonist VAF347 would be a good therapeutic candidate for diabetes and its complications, especially non-proliferative diabetic retinopathy.

## 4. Materials and Methods

### 4.1. Streptozotocin (STZ)-Induced Diabetic Mice

All experimental procedures were approved by CWRU IACUC (2016-0025 first approved on 2/13/2016) and LSCVAMC ACORP (16-012-MS-19-002-C first approved on 3/1/2016). Animals were housed under SPF conditions at Case Western Reserve University Animal Resource Center Health Sciences Animal Facility according to NIH guidelines. Male C57BL/6 mice aged 8 to 10 weeks were obtained from Jackson Laboratories (Bar Harbor, ME, USA). Diabetes was induced over five consecutive days with intraperitoneal injections of streptozotocin (STZ) at 60 mg/kg body weight. Diabetes was defined by 6-h, fasted-blood-glucose concentrations greater than 275 mg/dL, which was verified using glucose-dehydrogenase-based strips 17 days after the last STZ injection (day 22). Hyperglycemia was quantified by hemoglobin A1c levels using the Crystal Chem Mouse A1c kit at six and 29 weeks after diabetes was confirmed. Subcutaneous insulin (Eli Lilly NPH) was administered as needed (0 to 0.2 U, one to three times per week) to maintain body weight and prevent catabolism. Retinal inflammation, oxidative stress, and leukostasis analyses were performed at a two-month diabetic time point, while capillary degeneration analyses were performed at an eight-month diabetic time point. As previously described, these durations are optimal for these analyses in this murine model [23,26].

Briefly, the research design of this study was as follows: 30 mice per group (non-diabetic, VAF347-treated diabetic, and untreated diabetic mice), and 10 non-diabetic VAF347-treated (controls for VAF347 treatment toxicity) totaling 100 mice were used in this study; all HbA_1c_ scores are shown in Table 1. All 10 non-diabetic VAF347-treated mice were euthanized eight months after diabetes was confirmed for toxicity examination. Non-lethal blood collections of five mice per group were performed two and eight months after diabetes was confirmed for sera analysis of IL-6 and IL-17A. Two months after diabetes was confirmed, 20 mice/group (*n* = 60 mice total) were euthanized and used to analyze: the number of T helper cells in the spleen (*n* = 3 mice/group), the number of adherent leukocytes in the retinal vasculature (*n* = 3 mice/group), the level of oxidative stress in the retina (*n* = 5 mice/group), and the level of VEGF, IL-17A, IL-6, and TNF-α in the retina (*n* = 9 mice/group). Eight months after diabetes was confirmed, 10 mice/group (*n* = 30 mice total) were euthanized and used to analyze: the number of T helper cells in the spleen (*n* = 3 mice/group), the number of acellular capillaries in the retinal vasculature (*n* = 5 mice/group), and toxicity comparisons (*n* = 2 mice/group).

### 4.2. AhR Agonist VAF347 Treatment

VAF347((4-(3-Chloro-phenyl)-pyrimidin-2-yl)-(4-trifluoromethyl-phenyl)-amine) was purchased from Calbiochem. VAF347 is a cell-permeable, small-molecule agonist of the aryl hydrocarbon receptor (AhR). Directly targeting AhR binding, VAF347 controls the biological activity of AhR by interrupting signaling, while inhibiting dendritic cell maturation [20,21]. VAG539 is a water-soluble derivative of VAF347. In murine models of diabetes and asthma, orally administering 30 mg/kg of VAG539 inhibited dendritic cell maturation and proinflammatory cytokine production that would halt Th17 cell differentiation [21,22]; hence, we used 30 mg/kg of VAF347 in this injectable treatment regimen. Lyophilized VAF347 was suspended in DMSO and diluted in sterile saline to 30 mg/kg. Diabetic mice received weekly subcutaneous injections beginning one week after diabetes was confirmed (day 29). Mice analyzed at the two-month time point received 7 injections, while mice analyzed at the eight-month time point received 28 injections. Toxicity parameters of VAF347 in this model were defined by weekly measures of body weight, an assessment of body condition, and an analysis of lethargy and respiratory distress, as well as mortality and autopsy organ appearance. No toxicity was observed in any of the VAF347-treated mice.

### 4.3. CD4^+^ T Cell Isolation and IL-17A ELISA Analysis

Splenocytes were collected as previously described [41]. Briefly, individual spleens (*n* = 3 spleens/group) were removed, and a single cell suspension was generated and incubated in erythrocyte lysis buffer (eBioscience; ThermoFisher Scientific Waltham, MA, USA) for 5 min at 37 °C. CD4^+^ T cells were then isolated from the splenocytes by negative selection, using mouse CD4^+^ T cell enrichment columns (R&D Systems Minneapolis, MN, USA). Isolated T cells were >95% CD4^+^ whereas positivity was confirmed by flow cytometry analysis. After CD4^+^ positivity was confirmed, protein lysates were collected and analyzed using a two-site mIL-17A ELISA according to the manufacturer’s instructions (R&D Bioscience Minneapolis, MN, USA).

### 4.4. ELISA Analysis of Sera and Retina Protein

Retina protein lysates were collected and pooled from three retinas (*n* = 3 pooled samples of nine mice/group). Alternatively, sera were collected from five different mice for individual sample analysis (*n* = 5 samples/group). All retina lysate and sera samples were analyzed using a two-site mIL-6, mIL-17A, mTNF-a, and mVEGF ELISA according to the manufacturer’s directions (R&D Bioscience).

### 4.5. Staining of Retinal Vasculature for Leukostasis Analysis

Retinal vasculature was stained and leukostasis was analyzed as previously described [15,23]. Saline was perfused into the aorta to clear non-adherent leukocytes, then 10 mL of fluorescein-labeled concanavalin A lectin (1 mg/mL in PBS; Vector laboratories Burlington, ON Canada) was perfused to stain the retinal vasculature. After enucleation and isolation of the retina, flat mounts were imaged using a fluorescent stereoscope and the number of leukocytes adhered to the vasculature wall were counted (*n* = 3 samples/group). The 500 μm and 50 μm scale bar displayed gives a visual indicator of the size of the representative image.

### 4.6. Quantification of Oxidative Stress

Mice were perfused with 0.9% physiological saline; retinas were collected and incubated in Krebs-HEPES buffer (with 5 mmol/L glucose) for 25 min at 37 °C in 5% CO_2_. Luminescence was measured using Promega GLOMAX 20/20 luminometer at 5 min after the addition of 0.5 mmol/L of lucigenin, as previously described [11,42], to quantify the level of reactive oxygen species per retina (*n* = 5 retinas/group).

### 4.7. Retinal Capillary Degeneration Analysis

Acellular capillaries were quantified in the retinal vasculature, as previously described [10,26]. Enucleated globes were fixed with 10% formalin prior to retinal isolation. Retinas were incubated in elastase for 2 h, followed by acidic buffer overnight. Retinal vasculature was mechanically isolated and stained with hematoxylin and periodic acid-Schiff (*n* = 5/group). Acellular capillaries were quantified in seven field areas between the optic nerve and the periphery (200× magnification). Representative pictures were taken using a 40× objective mounted on an Olympus BX-60 microscope equipped with a Q-imaging Retiga Exi camera and Metamorph imaging software. The 10 μm scale bar displayed is a visual indicator of the size of the representative image.

### 4.8. Statistical Analysis

Statistical analysis was performed in Prism (GraphPad Software San Diego, CA, USA) using a two-way ANOVA and an unpaired *t*-test with Tukey’s post-hoc analysis. A *p*-value < 0.05 was considered significant.

## Figures and Tables

**Table 1:** Glycated Hemoglobin A_1c_ Data of Non-Diabetic (ND) and Diabetic (DB) Mice.

Group	%HbA_1c_ (Week 6)	%HbA_1c_ (Week 29)
C57BL/6-ND	5.2 ± 0.2	5.4 ± 0.4
C57BL/6-DB	11.5 ± 1.9 *	12.4 ± 2.3 *
+VAF347-ND	5.2 ± 0.3	5.2 ± 0.3
+VAF347-DB	11.4 ± 2.1 *	12.3 ± 1.5 *

Data are mean ± SD. * = *p* < 0.01 diabetic (DB) compared to non-diabetic (ND) per group.

## Data Availability

Data is available upon request to the corresponding author.
